# Crowding does not affect monarch butterflies’ resistance to a protozoan parasite

**DOI:** 10.1002/ece3.8791

**Published:** 2022-04-06

**Authors:** Wajd Alaidrous, Scott M. Villa, Jacobus C. de Roode, Ania A. Majewska

**Affiliations:** ^1^ 1371 Department of Biology Emory University Atlanta Georgia USA; ^2^ Division of Biological and Environmental Science and Engineering (BESE) King Abdullah University for Science and Technology Thuwal Saudi Arabia

**Keywords:** density‐dependent transmission, environmental transmission, host population density, host–parasite interaction, larval density

## Abstract

Host density is an important factor when it comes to parasite transmission and host resistance. Increased host density can increase contact rate between individuals and thus parasite transmission. Host density can also cause physiological changes in the host, which can affect host resistance. Yet, the direction in which host density affects host resistance remains unresolved. It is also unclear whether food limitation plays a role in this effect. We investigated the effect of larval density in monarch butterflies, *Danaus plexippus*, on the resistance to their natural protozoan parasite *Ophryocystis elektroscirrha* under both unlimited and limited food conditions. We exposed monarchs to various density treatments as larvae to mimic high densities observed in sedentary populations. Data on infection and parasite spore load were collected as well as development time, survival, wing size, and melanization. Disease susceptibility under either food condition or across density treatments was similar. However, we found high larval density impacted development time, adult survival, and wing morphology when food was limited. This study aids our understanding of the dynamics of environmental parasite transmission in monarch populations, which can help explain the increased prevalence of parasites in sedentary monarch populations compared to migratory populations.

## Graphical abstract

Crowding of hosts can have consequences on susceptibility and resistance to pathogens. In this study, we used the monarch butterfly and its protozoan to examine whether host density has an impact on infection probability and infection load when food is unlimited or limited. We found no effect on infection susceptibility or resistance in either food scenarios, yet strong effects on adult morphology with food limitation.

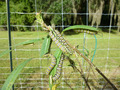

## 1 INTRODUCTION

Host density plays an important role in host–parasite interactions. For parasites that rely on direct contact between individuals for transmission, higher host density increases transmission and infection prevalence (Arneberg et al., 1998; Lloyd‐Smith et al., 2005; McCallum et al., 2001). Similarly, for parasites transmitted via the environment, increased host density can result in greater dissemination and accumulation of infectious stages in the environment and thereby increase incidence rates (Altizer et al., 2003; Arneberg et al., 1998). In parasites with complex life cycles, such as trematodes, production of infective stages is limited in time and space such that per capita host risk is diluted among all hosts (Buck & Lutterschmidt, 2017), resulting in a negative relationship between density and parasitism. Other work suggests that negative density‐dependent effects can occur in some host–parasite systems, particularly when hosts avoid infected individuals or areas with high transmission risk (Albery et al., 2020; Buck et al., 2018). Thus, the relationship between host density and infection risk is not always positive or straightforward.

Host density can impact susceptibility to parasitism, or the degree to which hosts are likely to become infected and experience subsequent parasite growth (Combes, 2001), although the underlying mechanism and direction of the relationships are often unclear (Michel et al., 2016). Hosts can decrease their susceptibility as density increases (i.e., density‐dependent prophylaxis) (Michel et al., 2016). For example, work on cabbage moths (*Mamestra brassicae*) (Goulson & Cory, 1995) and African armyworms (*Spodoptera exempta*) (Reeson et al., 1998) showed that larvae reared at higher densities had greater resistance to parasites, as measured by levels of melanization, a key part of insect immune function, among other protective functions (San‐Jose & Roulin, 2018). In contrast, other studies have shown that crowding increases intraspecific competition, aggression (Collie et al., 2020), and physiological stress (Steinhaus, 1958), supporting the crowding stress hypothesis. Crowding as a stress‐inducing factor for hosts can negatively impact host immune function (Lin et al., 2018; Michel et al., 2016; Steinhaus, 1958). For instance, grass carp (*Ctenopharyngodon idella*) long‐term crowding reduced immune parameters in the fish and their susceptibility to pathogens (Lin et al., 2018). Yet, other work found that crowding resulted in no changes in immunity (Adamo & Parsons, 2006). The complex interactions between host and parasite ecology at both the individual and community levels make predicting the influence of crowding on disease dynamics challenging.

Besides influencing transmission dynamics, crowding can also exacerbate the consequences of resource limitation and induce behavioral changes in hosts (Navarro et al., 2004). For instance, monarch butterfly caterpillars with low food quantity (milkweed leaves) were more aggressive toward conspecifics than those with higher food availability (Collie et al., 2020). More aggressive individuals likely expend more energy competing for resources, which may in turn reduce immunocompetence. However, food limitation in crowded environments also reduces food intake, which can impact host's ability to fight infection. Given that many wild animals are food limited and experience variable density environments, it is important to better understand how crowding interacts with food availability to influence host susceptibility.

The monarch butterfly, *Danaus plexippus*, and its parasite, *Ophryocystis elektroscirrha* (McLaughlin & Myers, 1970), provide a well‐suited system to study the effect of crowding on host susceptibility. *O*. *elektroscirrha* is a natural parasite that infects monarchs across their range (McLaughlin & Myers, 1970). Infection with *O*. *elektroscirrha* starts when a caterpillar ingests spores scattered onto eggs or plant leaves by adults (Altizer et al., 2004; de Roode et al., 2009). Transmission of the infection can occur via multiple routes. In addition to females transferring parasites to their eggs, both infected males and females can scatter spores on to milkweed. Moreover, infected males can transfer spore to females during mating, which they can then transmit to their offspring (Majewska et al., 2019). Parasites penetrate the mid‐gut wall, and infect the hypodermal tissues, where they replicate asexually and sexually during the larval and pupal stages. Adults emerge covered in millions of dormant spores (Leong et al., 1992; McLaughlin & Myers, 1970). Internal parasite growth is detrimental to monarchs, reducing survival to adulthood, mating success, fecundity, flight ability, and lifespan (Altizer & Oberhauser, 1999; Bradley & Altizer, 2005; de Roode et al., 2007, 2009).

Monarchs are known for their long‐distance migration from eastern North America to overwintering sites in Mexico (Brower, 1995; Reppert & de Roode, 2018; Urquhart & Urquhart, 1978). Recent decades have seen the formation of sedentary populations of monarchs, in mild climates of the southeastern USA, along the Gulf of Mexico, as well as in California, USA, where monarchs no longer migrate and breed year‐round on non‐native milkweed (Brower et al., 2012; Satterfield et al., 2015, 2016). Infection by *O*. *elektroscirrha* is more prevalent in sedentary than migratory monarch populations (Altizer et al., 2000; Satterfield et al., 2015, 2016), which is likely due to sedentary populations sustaining high host densities that breed all year‐round and, thus, experience higher parasite transmission (Altizer et al., 2004; Majewska et al., 2019). High caterpillar densities in sedentary populations have been associated with milkweed defoliation and food limitation (Fernández‐Haeger et al., 2015; Satterfield et al., 2016), potentially having detrimental effects on susceptibility, highlighting the need to explore the infection dynamics under these conditions.

Here, we examined the effect of larval density on host susceptibility to parasites in monarch butterflies (*Danaus plexippus*) in two experiments, one where food was unlimited and one where food was limited. Using the monarch's natural parasite *O*. *elektroscirrha*, we tested the effect of larval density on susceptibility and tolerance for the different treatment groups. In addition, we examined the effects of crowding on survival and development time of immature stages, as well as lifespan, wing size, and wing melanization of adults. Since larvae in higher densities are more likely to experience increased levels of physiological stress, we hypothesized that higher larval density would increase susceptibility to parasites, affecting developmental time and morphology.

## 2 METHODS

### 2.1 Caterpillar sources and rearing

We carried out two experiments to determine the effect of host density on disease susceptibility and tolerance. We used microcosms, which consisted of live potted plants, approximately 20–24 inches tall (50.8–61 cm) with two stalks, grown from seed in 4.5‐inch (11.43 cm)‐diameter pots contained within transparent plastic tubes (4 inch diameter × 24 inch height; 10.16 cm × 61 cm) and capped with netting. These microcosms were used to mimic natural conditions as closely as possible, with larvae experiencing crowding on live plants with minimal interference related to animal husbandry. All the larvae and plants used in this study were reared in a greenhouse. Lab‐reared monarchs were the breeding‐generation offspring of wild‐caught migrating North American monarch butterflies collected from St. Marks, Florida, USA (30.0737354°N, −84.1796806°W; a flyway and stopover site during the fall migration), in October 2017 and 2020, and overwintered in the laboratory. Mating and collection of eggs occurred in 0.6 m^3^ mesh cages. Larvae were randomly picked from four non‐inbred lineages for the larval densities treatments and all larvae were reared on *A*. *curassavica* for the duration of the experiments. This plant species was chosen specifically because it is the main species that monarchs in sedentary populations encounter in North America (Satterfield et al., 2015, 2016, 2018). Because the parasite and monarch lineages used for the two experiments differed and a significant amount of time passed following the first experiment (unlimited food experiment), we do not directly compare the outcomes of the two experiments and instead focus on the qualitative differences in the results.

### 2.2 Unlimited food experiment

In the first experiment, caterpillars had unlimited food supply and we asked whether rearing density influenced immature monarch survival, development, susceptibility, tolerance, lifespan, as well as adult wing size and melanization. Starting on day 2 of larval development, larvae were reared in microcosms in one of three density treatments: singles (1 caterpillar/plant), doubles (2 caterpillars/plant), or tens (10 caterpillars/plant). We provided larvae with new plants when necessary to ensure sustained food ad libitum. Our design was full factorial (for sample sizes, see Table 1). The singles treatment consisted of 25 replicates, doubles consisted of 15 replicates, and tens consisted of 6 replicates per inoculation treatments. Caterpillars in the inoculated treatment were individually inoculated with *O*. *elektroscirrha* parasites: second instar caterpillars were fed a 0.5 cm^2^ leaf disk of *A*. *curassavica* with 10 manually deposited spores (stain ID: E42‐2) in a Petri dish. Control caterpillars received a leaf disk without parasite spores. Upon complete consumption of their leaf disk, caterpillars were transferred to their randomly assigned microcosms. After pupation, pupae were transferred to individual 16 oz (473 ml) Solo cups and were attached to lids using hot glue. Placement of pupae in individual cups assured no cannibalism occurred in the high‐density treatment. Following emergence, adult monarchs were transferred to separate glassine envelopes without access to food and held in a DigiTherm^®^ incubator at 12°C.

**TABLE 1:** Number of monarchs used in each experiment along with percent of individuals surviving to adulthood and percent of infected adults in each treatment

Food unlimited						Food limited				
Inoculation treatment		Density treatment				Inoculation treatment		Density treatment		
		Singles	Doubles	Tens	Total			Singles	Tens	Total
Control	Initial number of caterpillars	25	30	59	114	Control	Initial number of caterpillars	25	58	83
	Number emerged	25	28	53	106		Number emerged	22	49	71
	% surviving to adulthood	100	93	90	93		% surviving to adulthood	88	84	86
	% infected	0	0	0	0		% infected	0	0	0
Inoculated	Initial number of caterpillars	25	30	60	115	Inoculated	Initial number of caterpillars	25	59	84
	Number emerged	22	29	58	109		Number emerged	22	45	67
	% surviving to adulthood	88	97	97	95		% surviving to adulthood	88	76	80
	% infected	91	93	98	95		% infected	73	73	73

### 2.3 Food limitation experiment

In the second experiment, we asked how density of monarchs per plant coupled with food limitation impacts immature monarch survival, development, susceptibility, tolerance, lifespan, wing size, and melanization of adult monarchs. We reared caterpillars in only two density treatments: singles (1 caterpillar/plant) and tens (10 caterpillars/plant). Because the first experiment revealed minimal effect of the two‐caterpillar density, and because of COVID‐19‐imposed research restrictions, this experiment did not include the doubles treatment. As before, our experimental design was full factorial. Second instar caterpillars in the inoculated treatment were inoculated with *O*. *elektroscirrha* parasites (strain ID E42 (P43)) and controls were fed parasite‐free leaf disks as described in the first experiment. To limit food availability, once all leaves in a microcosm were consumed, which only occurred in the 10‐caterpillar treatment, we provided one new plant. Next, on the second or third day of the fifth instar stage, we transferred the caterpillars from both density treatments to 16 oz Solo cups with *A*. *curassavica* plant stems (top portions of plant) only. Stems are often consumed by monarch caterpillars once supply of leaves is depleted and provide enough nutrition to complete development to pupation, while still ensuring food limitation (S. M. Villa unpub. data). As in the first experiment, all pupae were transferred to new individual 16 oz Solo cups and upon emergence adult monarchs were transferred to glassine envelopes and kept at 12°C in an incubator.

### 2.4 Survival, development time, and adult lifespan

We recorded death of caterpillars and pupae daily to measure immature survival. We noted larval and pupal development time by checking for pupation and eclosion once a day. Larval development time was quantified as the number of days from egg hatching to pupation, and pupal development time was quantified as the number of days from pupation to eclosion. We also calculated total development time as the sum of larval and pupal development times.

We checked the adults in the incubator daily until death, as routinely done in this experimental system (de Roode et al., 2007). We calculated lifespan as the number of days between eclosion and death. The lifespans obtained in this way closely mimic the lifespans of monarchs under more natural conditions (de Roode et al., 2009).

### 2.5 Susceptibility and tolerance

We measured host susceptibility via qualitative and quantitative resistance (Lefèvre et al., 2011). To estimate qualitative resistance, or the probability that monarchs became infected following inoculation, adult monarchs were tested for the presence or absence of parasites. We determined parasite spore load of adults in the inoculation treatment following de Roode et al. (2007). The abdomen of perished adults was removed and vortexed at maximum speed in 5 ml of tap water for 5 min. Next, we counted the number of spores present in 0.1 µl of the 5 ml suspension using a hemocytometer by averaging 16 chambers per sample. Monarchs with a spore load of zero were uninfected while those with spores were infected. Parasite spore load provides a measure of quantitative resistance, or the ability to limit parasite growth once infected, where higher load indicates higher susceptibility. We performed a log_10_ transformation on parasite spore loads for normality of error distributions and homogeneity of variance to meet model assumptions.

Finally, we estimated tolerance, the ability of the host to withstand increasing parasite load without a loss in fitness. We used adult monarch lifespan as a proxy for host fitness, which has been shown to be an important component of monarch fitness (de Roode et al., 2009). We examined the slopes of a linear relationship between adult lifespan and log_10_ parasite spore load for the three density treatments. Steeper reductions in adult lifespan with increasing parasite spore load indicate decreased tolerance (Lefèvre et al., 2011).

### 2.6 Wing size and melanization

To estimate wing area and wing melanization, we scanned the dorsal and ventral sides of the right wing with a Canon^®^ CanoScan LiDE 210 flatbed scanner and processed the images with ImageJ 1.52k (https://imagej.nih.gov/ij/). Briefly, we scanned wings at 300 dots per inch (dpi) to produce digital images for analysis. The scanner settings were constant for all individuals and no color correction was used. Wing analysis using scanned images has been widely used for analyzing monarch wing morphology (Davis, 2009; Davis et al., 2005, 2007, 2012; Hanley et al., 2013).

To process wing images, we first isolated the whole forewing and hindwing and quantified their area using the “measure” tool. Only the dorsal side of the wings was used for size to avoid redundancy. Adults with damaged wings were excluded. We then used a custom thresholding macro code to digitally separate the carotenoid‐based cells from the melanin‐based veins using the “thresholding” tool. Thresholding isolates the black from non‐black portions of the wings and has been used to previously analyze monarch wing color (Davis et al., 2005; Hanley et al., 2013).

We obtained melanization scores for all four wing surfaces (i.e., dorsal and ventral forewing and hindwing). The melanization score for each wing surface ranges from 0 (pure black) to 255 (pure white) and it is a measure of “blackness,” where lower values indicate more intense black coloration and greater melanin pigment in the wing. The four scores were then averaged to give an overall melanization score for each monarch. Previous work in lepidoptera suggests wing melanin pigmentation increases with immune function challenge (Freitak et al., 2005).

### 2.7 Statistical analysis

Statistical analysis was performed using R (R Core Team, 2021). We used generalized linear mixed‐effects models (GLMMs) with binomial errors to test for differences in immature survival (0: perished; 1: alive) and infection status (0: uninfected; 1: infected) between density treatments. Fixed effects in the survival model included density and inoculation treatment, while in the infection model fixed effects included density and sex. We did not include sex in the analysis of immature survival because sex is unknown until adulthood. We used linear mixed‐effects models (LMM) with Gaussian errors to test for differences in development times (larval, pupal, and total development), adult lifespan, and parasite spore load between density treatments. To assess whether parasite spore load differed between density treatments we used a LMM with fixed effects as before: density, inoculation, a density‐by‐inoculation interaction, and sex. In all models, the unique microcosm that the larvae were reared in was included as a random effect.

To examine the differences in tolerance between density and inoculation treatments, we employed a LMM with adult lifespan as the response variable, and sex, log_10_ spore load, density, and the interaction between log_10_ spore load and density as explanatory factors.

Finally, we asked whether wing morphology varies with density and inoculation treatments. LMMs were used to compare wing areas and wing melanization across the density treatments. Fixed effects included density, inoculation, the interaction between density and inoculation treatments and sex. The microcosm that the larvae were reared in was included as a random effect as before.

## 3 RESULTS

### 3.1 Unlimited food experiment

#### 3.1.1 Survival, development time, and adult lifespan

**FIGURE 1:**
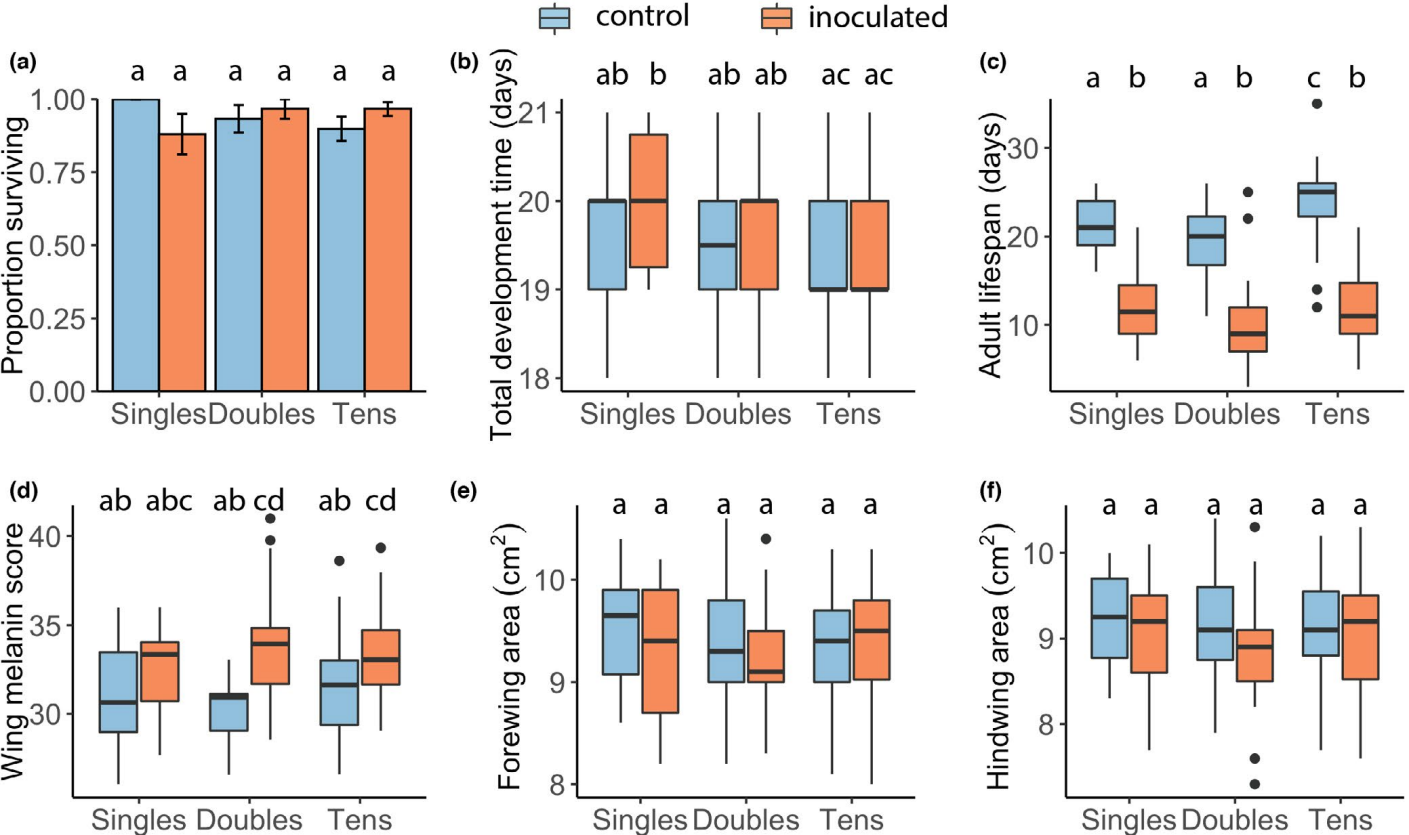
Density and inoculation treatment in relation to (a) proportion of surviving immature monarchs to adulthood, (b) total development time, (c) adult lifespan, (d) wing melanin score, (e) forewing, and (f) hindwing area in the unlimited food experiment. Bars represent means, color of bars represent treatment (blue: control; orange: inoculated), and error bars represent standard errors of the mean. Box plots show median values (thick black middle lines) with first and third quartiles (boxes), maximum and minimum values (whiskers), and outliers (black points). Different letters above box plots indicate significant differences (Table S7–S12)

Immature survival probabilities tended to be high (above 90%, Table 1) and did not significantly differ among density and inoculation treatments (*p *> .05, Table 2, Figure 1a). We found no impact of inoculation, density treatment, or their interaction on larval, pupal, or total development times (*p *> .05; Figure 1b). Sex significantly impacted development: males had longer larval, pupal, and total development times than females (larval: *t* = 1.99, *p* = .05; pupal: *t* = 9.01, *p* < .001; and total: *t* = 7.32, *p* < .001).

**TABLE 2:** Summary of the variables included in the two experiments and model results

Response variable		Fixed effect				
		Density (Single/Doubles/Tens)	Inoculation (Inoculated/Control)	Density × Inoculation	Sex (M/F)	Spore load
Unlimited food	Immature survival (0/1)	ns	ns	*//*	//	//
	Larval development time	ns	ns	ns	M*	//
	Pupal development time	ns	ns	ns	M***	//
	Total development time	ns	ns	ns	M***	//
	Adult lifespan	Tens**	Inoculated***	Tens × Inoculated**	M**	//
	Forewing area	ns	ns	ns	ns	//
	Hindwing area	ns	ns	ns	M*	//
	Melanin score	ns	ns	Doubles × Inoculated**	M*	//
	Infection (0/1)	ns	*//*	*//*	ns	//
	Spore load	ns	*//*	*//*	ns	//
	Tolerance	ns	ns	ns	M***	***

	Response variable	Density (Single/Tens)	Inoculation (Inoculated/Control)	Density x Inoculation	Sex (M/F)	Spore load
Food limited	Immature survival (0/1)	ns	Ns	*//*	*//*	*//*
	Larval development time	Tens***	Ns	ns	M***	*//*
	Pupal development time	ns	Ns	ns	M***	*//*
	Total development time	Tens***	Ns	ns	M***	*//*
	Adult lifespan	Tens***	Inoculated***	Tens x Inoculated *	ns	*//*
	Forewing area	Tens***	ns	ns	ns	*//*
	Hindwing area	Tens***	ns	ns	M*	*//*
	Melanin score	Tens***	Inoculated***	ns	ns	*//*
	Infection (0/1)	ns	ns	*//*	*//*	*//*
	Spore load	Tens*	ns	ns	ns	*//*
	Size‐corrected spore load	ns	ns	ns	ns	*//*
	Tolerance	ns	ns	ns	ns	ns

Note

Fixed effects were density, inoculation treatment, interaction between density and inoculation treatment, and sex. Microcosm identification was included as a random effect in all models. Each row summarizes a model for a different response variable. “ns” represents a non‐significant term and “//” indicates that the variable was not included in the model. Asterisks denote the *p*‐value, *p < .05, **p < .01; ***p < .001. For model results, see Appendix Tables S1–S6.

Density treatment significantly impacted adult lifespan: monarchs in the 10‐caterpillar treatment had longer lifespan compared to those in singles and doubles densities (*t* = 2.84, *p* = .01). Inoculation treatment had a strong impact on lifespan: compared to inoculated monarchs, control monarchs lived about twice as long (*t* = −7.67, *p *< .001; Figure 1c). Sex also impacted lifespan: males lived significantly less time than females (*t* = −3.04, *p* < .01; Table 2). Finally, we found a significant interaction between density and inoculation treatments: monarchs in the n 10‐caterpillar inoculated treatment combination showed significantly shorter (nearly half as long) adult lifespan compared to other treatment combinations (*t* = −2.00, *p* = .05; Figure 1c).

#### 3.1.2 Wing size and melanization

We found no effect of density, inoculation treatments, or their interaction on wing area when food was unlimited (*p *> .05, Table 2; Figure 1e,f). Sex significantly impacted hindwing size: males had slightly larger hindwings than females (*t* = 2.14, *p* = .04; Table 2). Melanin score was significantly impacted by the interaction between inoculation and density (Figure 1d) as well as sex: adults in the double inoculated treatment had somewhat higher melanin scores (i.e., less black density; *t* = 2.14, *p* = .03) while males showed slightly lower melanin scores (i.e., greater black density; *t* = −2.49, *p* = .01).

#### 3.1.3 Susceptibility and tolerance

**FIGURE 2:**
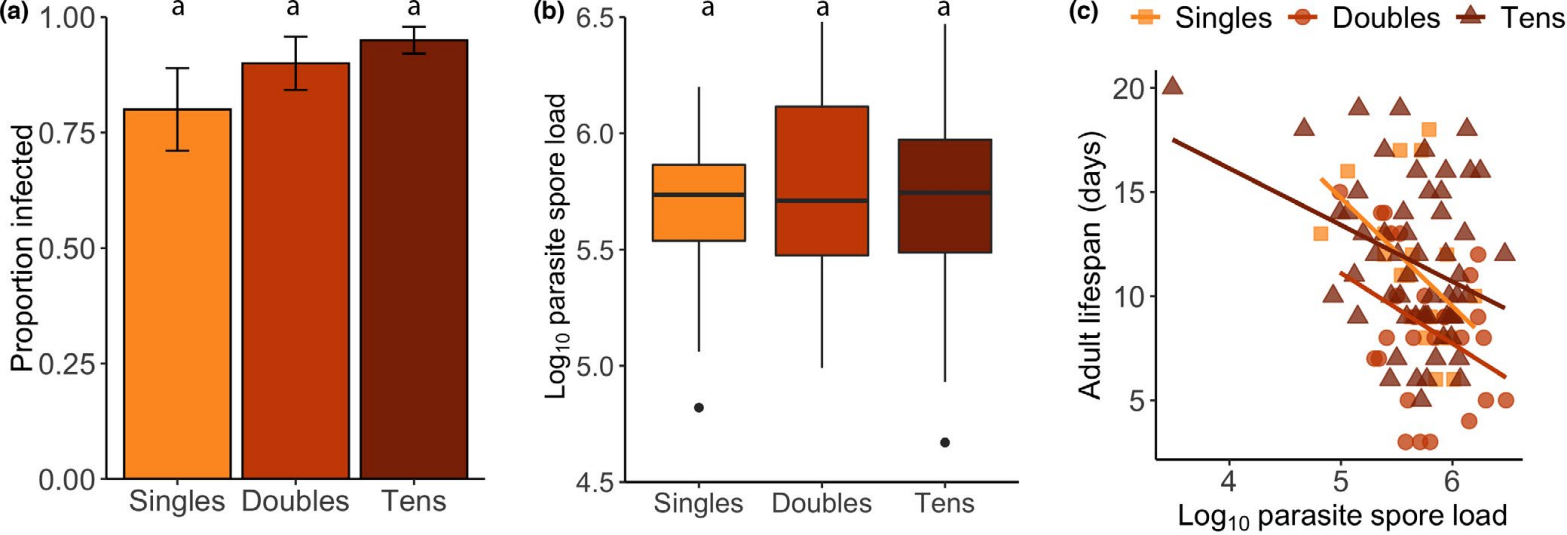
Effect of density treatment (singles, doubles, and tens) in relation to (a) proportion of monarchs that became infected in the inoculated treatment, and (b) log_10_ parasite spore load and (c) tolerance (the slope of the relationship between adult lifespan and parasite spore load) in the unlimited food experiment. Bars represent means, and error bars represent standard errors of the mean. Color of bars, points, and lines represent density treatment (light orange: singles; orange: doubles; and dark orange: tens). Box plots show median values (thick black middle lines) with first and third quartiles (boxes), maximum and minimum values (whiskers), and outliers (black points). Different letters above box plots indicate significant differences (Table S13–S14)

We found that 95% of the adults in the inoculation treatment became infected (singles: 91%, doubles: 93%, and tens: 98%; Table 1). Several caterpillars and pupae died prior to end of both experiments due to observer error (e.g., accidental physical damage) and unknown causes. Probability of infection (qualitative resistance) did not significantly differ across the density treatments (*p* > .05; Table 2; Figure 2a). Analysis of the infected adults only showed no effect of density on parasite spore load (quantitative resistance; *p* > .05; Table 2; Figure 2b). Adult lifespan was negatively affected by parasite spore load (*t* = −3.45, *p* < .001), but not by density (*p* > .05; Table 2; Figure 2c). We found no significant interaction between spore load and density on lifespan (*p* > .05), indicating no overall differences in tolerance between density treatments. For model outputs, see Appendix Table S3.

### 3.2 Food limitation experiment

#### 3.2.1 Survival, development time, and adult lifespan

**FIGURE 3:**
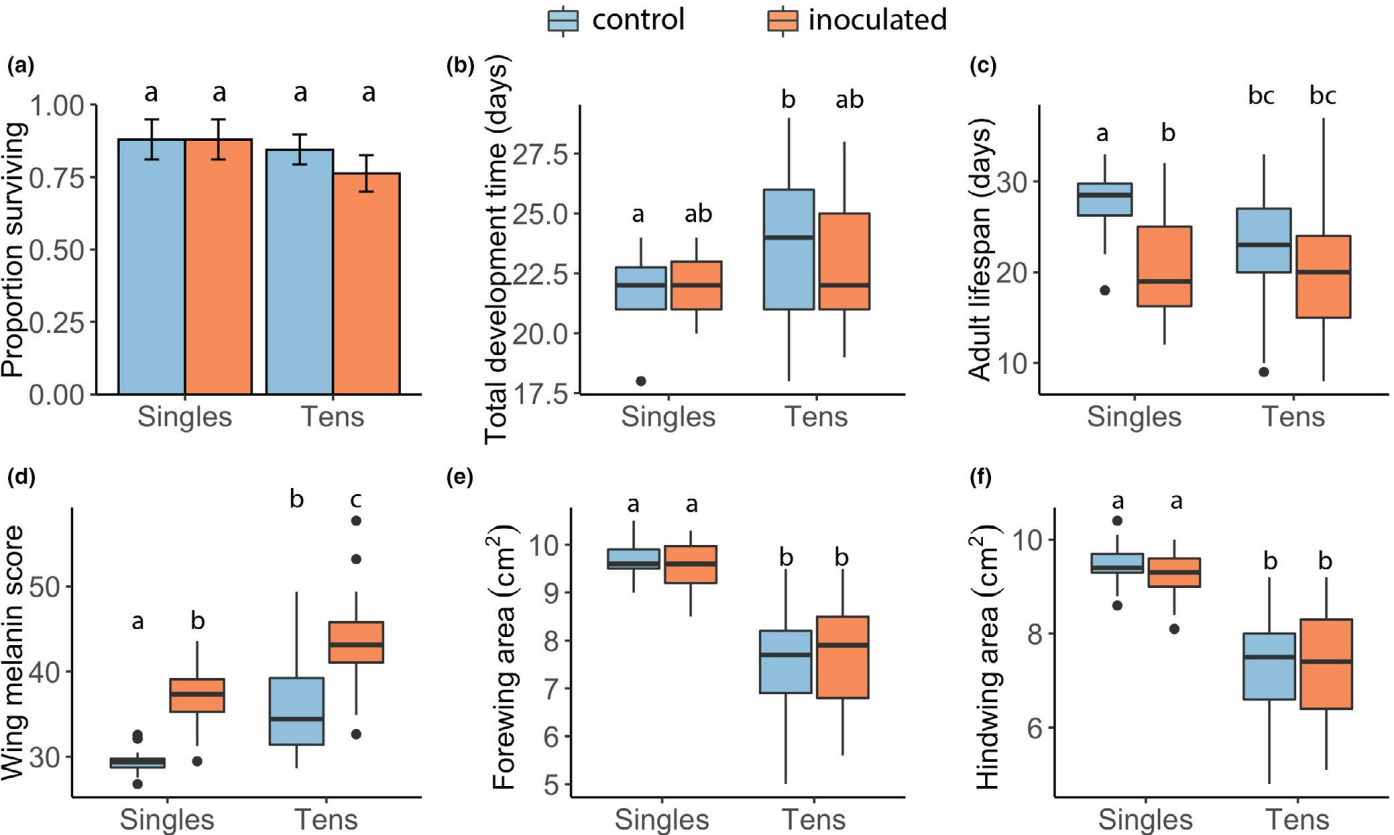
Density and inoculation treatment in relation to (a) proportion of surviving immature monarchs to adulthood, (b) total development time, (c) adult lifespan, (d) wing melanin score, (e) forewing, and (f) hindwing area in the food limitation experiment. Bars represent means, color of bars represent treatment (blue: control; orange: inoculated), and error bars represent standard errors of the mean. Different letters above box plots indicate significant differences (Table S15–S20)

When food was limited, survival to adulthood tended to decrease among inoculation treatments but this difference was not statistically significant (*p *> .05; Table 2); we also found no significant difference in survival between the singles and tens density treatments (*p *> .05; Figure 3a).

Density but not inoculation affected larval and total development times: caterpillars in the high‐density treatment (tens) took significantly longer to develop than those in the singles treatment (larval: *t* = 3.4, *p* = .001; total: *t* = 2.70, *p* = .01; Figure 3b). Inoculation and density treatments did not impact pupal development time (*p *> .05). We found that sex affected development times, with males showing longer larval (*t* = 3.32, *p* = .001), pupal (*t* = 4.78, *p *< .001), and total development (*t* = 4.17, *p* < .001) times compared to females (Table 2). We found no effect of the interaction between inoculation and density treatment on development times (*p *> .05).

Adult lifespan was significantly affected by density and inoculation treatments when food was limited for caterpillars. Monarchs in higher density (tens) had slightly shorter lifespan (*t* = −3.13, *p* < .01) than those in single densities, and those in inoculated treatment lived shorter than controls (*t* = −4.05, *p* < .001; Figure 3c). We also found a significant interaction between density and inoculation treatments: monarchs in the 10‐caterpillar inoculated treatment combination showed significantly shorter adult lifespan compared to other density inoculation treatment combinations (t = 2.08, *p* = .04; Figure 3c).

#### 3.2.2 Wing size and melanization

Density but not inoculation impacted wing size when food was limited: both forewing and hindwing areas were significantly smaller in the tens density treatment (forewing: *t* = −8.95, *p* < .001; hindwing: *t* = −9.07, *p* < .001; Figure 3e,f). We found no effect of the interaction between inoculation and density treatment on wing areas (*p *> .05). Sex impacted hindwing but not forewing area: males had significantly larger hindwings compared to females (*t* = 2.09, *p* = .04). Melanin score was impacted by density and inoculation treatments but not sex (Table 2). Monarchs in the tens density treatment had higher melanin scores compared to singles treatments (*t* = 5.30, *p* < .001). Similarly, inoculated monarchs had higher melanin scores compared to controls (*t* = 5.80, *p* < .001; Figure 3d). We found no effect of the interaction between inoculation and density treatment on the melanin score (*p* > .05).

#### 3.2.3 Susceptibility and tolerance

**FIGURE 4:**
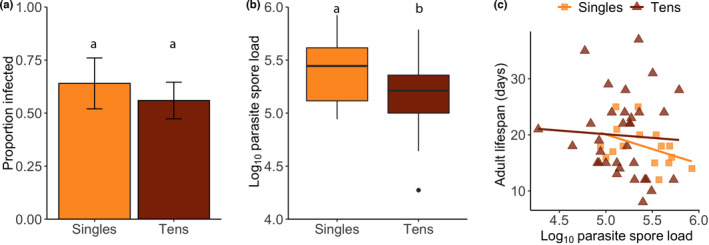
Density in relation to (a) proportion of monarchs that became infected in the inoculated treatment, (b) log_10_ parasite spore load, and (c) tolerance (the slope of the relationship between adult lifespan and parasite spore load) in the food limitation experiment. Bars represent means, and error bars represent standard errors of the mean. Color of bars, points, and lines represent density treatment (light orange: singles; dark orange: tens). Different letters above box plots indicate significant differences (Table S21–S22)

A total of 73% of the adults in the inoculation treatment became infected (singles: 73%, tens: 73%; Table 1). Infection probability (qualitative resistance) was not impacted by density treatment (*p *> .05; Figure 4a). Analysis of the infected adults only showed that monarchs in the 10‐caterpillar treatment had lower parasite spore loads compared to singles (quantitative resistance; *t* = −2.10, *p* = .05; Figure 4b). Because both parasite growth and monarch size could be affected by crowding, and given the smaller size of infected monarchs (see above), we followed up with an analysis of parasite spore load corrected for wing size (residuals of a simple linear regression between wing area and spore load). Examination of the corrected spore load in relation to density showed no significant differences across the density treatments (*p *> .05; Table 2). Neither spore load nor density nor the interaction of the two influenced adult lifespan indicating that density did not alter tolerance of infection (*p *> .05; Figure 4c). For model outputs, see Appendix Tables S3–S6.

## 4 DISCUSSION

In this study, we examined the effect of crowding and food availability at larval stages on disease susceptibility in monarch butterflies. When food was unlimited, high density had no effect on infection probability (qualitative resistance), parasite load (quantitative resistance), or tolerance. Under food‐limited conditions, crowding also did not impact the probability of infection, yet monarchs reared in the highest density (10‐caterpillar treatment) had a lower parasite load than those reared at the lowest density (single‐caterpillar treatment), suggesting that high rearing density lowers caterpillar parasite susceptibility. On the other hand, lower parasite load among the hosts held at high density might be a consequence of the starvation and small host size (Pulkkinen & Ebert, 2004). Indeed, accounting for wing size, we found no significant differences in spore load between density treatments. It is also important to consider that the food type (leaves vs. stem) that caterpillars consumed under high‐density conditions might have impacted the parasite load, and experiments examining this possibility are needed.

Interestingly, we found that in both experiments, infected monarchs showed less dense wing melanization (i.e., higher scores). Since melanin is costly to produce, these results suggest that the energetic costs of *O*. *elektroscirrha* reduce a monarch's “blackness.” Moreover, less melanin production might also suggest a lack of resources to mount an effective immune defense (Freitak et al., 2005). Since melanization is considered a signal of immunocompetence in insects (Nakhleh et al., 2017; Wilson et al., 2001), the differential wing melanization among infected individuals might be an honest signal of monarch health and quality. We also found an effect of food availability on wing melanin. When food was unlimited, there was no significant difference within infection treatments among singles, doubles, and tens. However, when food was limited, wing melanization was less dense for both infected and uninfected monarchs when raised in the tens treatment compared to the singles treatment. This suggests that less food also restricts a monarch's ability to produce melanin. Thus, both food availability and parasites can additively influence monarch melanization. Furthermore, consumption of milkweed stems only at high densities might affect melanization, although this was not tested in this study. Interestingly, the darkest monarchs in our experiments were uninfected singles with unlimited food, and the least melanized ones were infected tens with limited food. Future work should assess immune parameters in monarchs under varying densities, food availability and type (stem vs. leaves), and infection status to better understand the relationships between wing melanization and immunity in this species.

Our results are in contrast with a previous study that suggested that crowding caused increased infection probability in monarchs (Lindsey et al., 2009). However, differences in methodology and milkweed species used between our study and the Lindsey et al. (2009) experiment make direct comparisons of findings difficult. In particular, Lindsey et al. (2009) raised caterpillars on cuttings of *A*. *incarnata* rather than live plants of *A*. *curassavica*. The quick deterioration of milkweed cuttings combined with the buildup of frass on plant material necessitated frequent handling of the caterpillars which likely increased the stress of the caterpillars in the high‐density treatment compared to the study we described here. Furthermore, caterpillars in Lindsey et al. (2009) study experienced other stressors, including an unidentified viral or bacterial disease that caused high mortality and might have influenced the outcomes.

The finding that crowding in our experiments did not increase monarch susceptibility to infection does not mean that higher density will lessen disease pressure in natural monarch populations. Instead, we expect the effects of crowding to affect parasite transmission. Theory suggests that diseases that spread via density‐dependent transmission show increased parasite prevalence with crowding due to increased contact rates between hosts (McCallum et al., 2001; Rader et al., 2020). Moreover, higher densities can result in greater buildup of infectious parasite stages in the environment, and thereby result in greater infection rates (Arneberg et al., 1998; Majewska et al., 2019). Both of these factors are highly relevant to monarch butterflies, some of which are foregoing migration to form sedentary populations to breed year‐round in North America (Satterfield et al., 2015, 2016). The high densities characterized by sedentary populations have been associated with increased parasite prevalence, most likely because of greater exchange of parasites between adults and greater deposition of spores onto milkweed foliage (Majewska et al., 2019; Satterfield et al., 2015). Given our results, it is unlikely that the patterns observed in the field are driven by increased susceptibility, but instead driven by greater transmission rates. As more migratory monarchs switch to sedentary lifestyles, it becomes increasingly important to study infection dynamics in sedentary populations and the role of lost migration in shaping parasite transmission. This study enhances our understanding of the infection transmission dynamics in monarch populations and possible causes for the increase in parasite prevalence in sedentary monarchs.

Food is rarely unlimited in nature and crowding is likely to increase intraspecific competition and, in turn, physiological and resource stress, all of which can negatively impact life history traits (Boggs, 2009). Not surprisingly, when food was limited, fewer monarchs survived to adulthood compared to when food was unlimited. Furthermore, crowded and food‐limited monarch caterpillars developed more slowly into adults and experienced shorter adult lifespans than monarchs raised singly. Crowding coupled with food limitation also caused reductions in wing size and less dense melanin (i.e., less “blackness”) in the wings. All effects observed here are consistent with numerous other studies examining the influence of crowding on life history traits in insects (Alto et al., 2012; Baldal et al., 2005; Banks & Thompson, 1987; Gibbs et al., 2004; Scheiring et al., 1984).

The impact of food limitation on monarchs is particularly noticeable when comparing the results of our unlimited and limited food experiments: when food was unlimited, crowding had no effect on developmental rate or wing size, yet food limitation led to longer developmental times and smaller wing size. These findings are consistent with previous work in monarchs (e.g., Johnson et al., 2014). In another study on the effects of larval rearing density in monarchs, larvae showed similar developmental times in high density and constant food supply (Atterholt & Solensky, 2010). Yet, in our study, the highest‐density treatment had a higher number of individuals (*n* = 10 caterpillars), which suggests that starvation and high levels of crowding have a strong effect on development time. Atterholt and Solensky (2010) found no effect of starvation on monarch size, or development time when monarchs were raised singly. However, Atterholt and Solensky (2010) imposed food stress by removing larvae from their food source at certain intervals and this method might not have been effective at imposing food stress. Furthermore, survival to adulthood has been shown to decrease with increasing egg per plant density (Nail et al., 2015). Thus, crowding at very high densities can have more pronounced effects on survival in nature, where additional factors such as the presence of predators are likely impacting survival.

In conclusion, our experiments revealed that monarch butterfly susceptibility and tolerance to a protozoan parasite tends to be similar across varying caterpillar densities and we found no evidence for the crowding stress hypothesis or density‐dependent prophylaxis hypothesis in this system. Nonetheless, we note that under certain ecological scenarios, crowding can strongly impact other key traits, including development time, adult lifespan, and wing melanization, all of which might have consequences for the persistence of healthy monarch populations. The biggest impact of crowding may be found in altering transmission rates in monarchs, and future work should directly test this prediction.

## CONFLICT OF INTEREST

We declare no conflicts of interest.

## AUTHOR CONTRIBUTIONS

**Wajd Alaidrous:** Conceptualization (lead); Data curation (lead); Formal analysis (supporting); Investigation (lead); Methodology (lead); Project administration (lead); Visualization (supporting); Writing – original draft (lead); Writing – review & editing (supporting). **Scott M. Villa:** Data curation (supporting); Formal analysis (supporting); Methodology (supporting); Software (supporting); Writing – review & editing (supporting). **Jacobus C. de Roode:** Conceptualization (supporting); Data curation (supporting); Formal analysis (supporting); Funding acquisition (lead); Investigation (supporting); Methodology (supporting); Project administration (supporting); Resources (lead); Supervision (lead); Writing – original draft (supporting); Writing – review & editing (supporting). **Ania A. Majewska:** Data curation (supporting); Formal analysis (lead); Software (supporting); Supervision (supporting); Validation (lead); Visualization (supporting); Writing – original draft (supporting); Writing – review & editing (lead).

## Supporting information

Supplementary Material

## Data Availability

Data are publicly available and archived in Dryad (Alaidrous et al., 2022): https://doi.org/10.5061/dryad.w0vt4b8tk.
