# Supplementary material for: Crowding does not affect monarch butterflies’ resistance to a protozoan parasite
Source: Ecol Evol. 2022 Apr 6;12(4):e8791. doi: 10.1002/ece3.8791 (PMC8986514; doi:10.1002/ece3.8791)
Supplement: Supplementary file 1 — Supplementary Material [file ECE3-12-e8791-s001.docx]

**Appendix**

To accompany **Crowding does not affect monarch butterflies’ resistance to a protozoan parasite**

by Wajd Alaidrous, Scott M. Villa, Jacobus C. de Roode, Ania A. Majewska

Corresponding author: Ania A. Majewska, ania.majewska@emory.edu

**Table S1**. Results of models investigating the effect of larval density on (a) survival, (b) development time, and lifespan in the unlimited food experiment. Microcosm was included as a random effect in all linear models. Significant effect sizes (P-value ≤ 0.05) appear in bold.

a)

| **Response Variable** | **Fixed Effect** | **Estimate (SE)** | **z value** | **P-value** |
| --- | --- | --- | --- | --- |
| Immature survival (0/1) | Density: Doubles | 0.20 (0.86) | 0.23 | 0.82 |
|  | Density: Tens | -0.23 (0.72) | -0.32 | 0.75 |
|  | Inoculation: Inoculated | 0.36 (0.65) | 0.55 | 0.58 |

b)

| **Response Variable** | **Fixed Effect** | **Estimate (SE)** | **t value** | **d.f.** | **P-value** |
| --- | --- | --- | --- | --- | --- |
| Larval development time | Density: Doubles | -0.17 (0.15) | -1.14 | 128.32 | 0.26 |
|  | Density: Tens | -0.22 (0.14) | -1.51 | 30.79 | 0.14 |
|  | Inoculation: Inoculated | 0.30 (0.15) | 1.93 | 180.99 | 0.06 |
|  | **Sex: Male** | **0.14 (0.07)** | **1.99** | **199.50** | **0.05** |
|  | Density: Doubles x Inoculation: Inoculated | -0.14 (0.21) | -0.66 | 131.77 | 0.51 |
|  | Density: Tens x Inoculation: Inoculated | -0.25 (0.21) | -1.22 | 32.66 | 0.23 |
| Pupal development time | Density: Doubles | 0.06 (0.12) | 0.47 | 121.88 | 0.64 |
|  | Density: Tens | -0.07 (0.11) | -0.61 | 19.14 | 0.55 |
|  | Inoculation: Inoculated | 0.06 (0.12) | 0.48 | 187.12 | 0.63 |
|  | **Sex: Male** | **0.53 (0.06)** | **9.01** | **199.93** | **<0.001** |
|  | Density: Doubles x Inoculation: Inoculated | -0.14 (0.17) | -0.81 | 125.95 | 0.42 |
|  | Density: Tens x Inoculation: Inoculated | -0.14 (0.16) | -0.89 | 20.55 | 0.38 |
| Total development time | Density: Doubles | -0.11 (0.18) | -0.62 | 170.86 | 0.54 |
|  | Density: Tens | -0.28 (0.17) | -1.68 | 52.00 | 0.10 |
|  | Inoculation: Inoculated | 0.36 (0.19) | 1.83 | 198.23 | 0.07 |
|  | **Sex: Male** | **0.68 (0.09)** | **7.32** | **201.58** | **<0.001** |
|  | Density: Doubles x Inoculation: Inoculated | -0.28 (0.27) | -1.03 | 173.10 | 0.30 |
|  | Density: Tens x Inoculation: Inoculated | -0.39 (0.24) | -1.58 | 55.19 | 0.12 |
| Adult lifespan | Density: Doubles | -1.61 (1.13) | -1.42 | 130.76 | 0.16 |
|  | **Density: Tens** | **3.10 (1.09)** | **2.84** | **31.77** | **0.01** |
|  | **Inoculation: Inoculated** | **-9.03 (1.18)** | **-7.67** | **181.82** | **<0.001** |
|  | **Sex: Male** | **-1.68 (0.55)** | **-3.04** | **197.53** | **<0.01** |
|  | Density: Doubles x Inoculation: Inoculated | -0.50 (1.64) | -0.31 | 134.17 | 0.76 |
|  | **Density: Tens x Inoculation: Inoculated** | **-3.14 (1.57)** | **-2.00** | **33.44** | **0.05** |

**Table S2.** Results of models investigating the effect of larval density on wing areas (forewing and hindwing), and melanin score in the unlimited food experiment. Microcosm was included as a random effect in all linear models. Significant effect sizes (P-value ≤ 0.05) appear in bold.

| **Response Variable** | **Fixed Effect** | **Estimate (SE)** | **t value** | **d.f.** | **P-value** |
| --- | --- | --- | --- | --- | --- |
| Forewing area | Density: Doubles | -0.10 (0.17) | -0.60 | 120.67 | 0.55 |
|  | Density: Tens | -0.18 (0.17) | -1.05 | 40.81 | 0.30 |
|  | Inoculation: Inoculated | -0.16 (0.17) | -0.97 | 163.11 | 0.33 |
|  | Sex: Male | 0.10 (0.08) | 1.37 | 187.93 | 0.17 |
|  | Density: Doubles x Inoculation: Inoculated | 0.01 (0.24) | 0.06 | 123.85 | 0.95 |
|  | Density: Tens x Inoculation: Inoculated | 0.28 (0.24) | 1.14 | 42.01 | 0.26 |
| Hindwing area | Density: Doubles | -0.08 (0.18) | -0.43 | 129.49 | 0.66 |
|  | Density: Tens | -0.13 (0.18) | -0.71 | 46.79 | 0.48 |
|  | Inoculation: Inoculated | -0.20 (0.18) | -1.06 | 168.39 | 0.29 |
|  | **Sex: Male** | **0.18 (0.08)** | **2.14** | **189.21** | **0.04** |
|  | Density: Doubles x Inoculation: Inoculated | -0.14 (0.26) | -0.56 | 132.51 | 0.57 |
|  | Density: Tens x Inoculation: Inoculated | 0.11 (0.26) | 0.43 | 48.13 | 0.67 |
| Melanin score | Density: Doubles | -0.66 (0.73) | -0.90 | 124.38 | 0.37 |
|  | Density: Tens | 0.66 (0.74) | 0.89 | 40.47 | 0.38 |
|  | Inoculation: Inoculated | 1.38 (0.76) | 1.81 | 166.27 | 0.07 |
|  | **Sex: Male** | **-0.86 (0.35)** | **-2.49** | **187.10** | **0.01** |
|  | **Density: Doubles x Inoculation: Inoculated** | **2.28 (1.07)** | **2.14** | **128.01** | **0.03** |
|  | Density: Tens x Inoculation: Inoculated | 0.40 (1.07) | 0.37 | 42.49 | 0.71 |

**Table S3.** Results of models investigating the effect of larval density on infection status, spore load, and tolerance in the unlimited food experiment. Microcosm was included as a random effect in all linear models. Significant effect sizes (P-value ≤ 0.05) appear in bold.

a)

| **Response Variable** | **Fixed Effect** | **Estimate (SE)** | **z value** | **P-value** |
| --- | --- | --- | --- | --- |
| Infection (0/1) | Density: Doubles | 0.30 (1.04) | 0.29 | 0.77 |
|  | Density: Tens | 1.74 (1.25) | 1.39 | 0.16 |
|  | Sex: Male | -1.43 (1.15) | -1.25 | 0.21 |

b)

| **Response Variable** | **Fixed Effect** | **Estimate (SE)** | **t value** | **d.f.** | **P-value** |
| --- | --- | --- | --- | --- | --- |
| Spore load | Density: Doubles | 0.11 (0.13) | 0.80 | 84.02 | 0.43 |
|  | Density: Tens | -0.03 (0.12) | -0.22 | 39.00 | 0.83 |
|  | Sex: Male | -0.14 (0.09) | -1.66 | 92.01 | 0.10 |
| Tolerance  (adult lifespan) | **Spore load** | **-7.79 (2.61)** | **-3.45** | **83.70** | **<0.001** |
|  | Density: Doubles | -17.83 (15.95) | -1.12 | 80.72 | 0.26 |
|  | Density: Tens | -25.32 (13.73) | -1.84 | 86.38 | 0.07 |
|  | Spore load x Density: Doubles | 2.76 (2.79) | 0.99 | 80.82 | 0.33 |
|  | Spore load x Density: Tens | 4.51 (2.42) | 1.87 | 86.56 | 0.07 |
|  | **Sex: Male** | **-3.31 (0.64)** | **-5.16** | **86.63** | **<0.001** |

**Table S4.** Results of models investigating the effect of larval density on (a) survival, (b) development time, and lifespan in the food limitation experiment. Microcosm was included as a random effect in all linear models. Significant effect sizes (P-value ≤ 0.05) appear in bold.

a)

| **Response Variable** | **Fixed Effect** | **Estimate (SE)** | **z value** | **P-value** |
| --- | --- | --- | --- | --- |
| Immature survival (0/1) | Density: Tens | -0.61 (0.54) | -1.13 | 0.26 |
|  | Inoculation: Inoculated | -0.37 (0.49) | -0.76 | 0.45 |

b)

| **Response Variable** | **Fixed Effect** | **Estimate (SE)** | **t value** | **d.f.** | **P-value** |
| --- | --- | --- | --- | --- | --- |
| Larval development time | **Density: Tens** | **1.83 (0.54)** | **3.40** | **61.77** | **0.001** |
|  | Inoculation: Inoculated | 0.51 (0.59) | 0.87 | 130.02 | 0.38 |
|  | **Sex: Male** | **1.04 (0.33)** | **3.32** | **132.96** | **0.001** |
|  | Density: Tens x Inoculation: Inoculated | -1.46 (0.77) | -1.90 | 62.67 | 0.06 |
| Pupal development time | Density: Tens | -0.08 (0.18) | -0.47 | 51.17 | 0.64 |
|  | Inoculation: Inoculated | -0.15 (0.20) | -0.78 | 129.81 | 0.44 |
|  | **Sex: Male** | **0.53 (0.11)** | **4.78** | **133.00** | **<0.001** |
|  | Density: Tens x Inoculation: Inoculated | 0.18 (0.25) | 0.69 | 51.89 | 0.49 |
| Total development time | **Density: Tens** | **1.75 (0.65)** | **2.70** | **59.47** | **0.01** |
|  | Inoculation: Inoculated | -0.36 (0.70) | -0.52 | 128.85 | 0.61 |
|  | **Sex: Male** | **1.63 (0.39)** | **4.17** | **132.83** | **<0.001** |
|  | Density: Tens x Inoculation: Inoculated | -1.28 (0.93) | -1.38 | 60.42 | 0.17 |
| Adult lifespan | **Density: Tens** | **-4.67 (1.50)** | **-3.13** | **133.00** | **<0.01** |
|  | **Inoculation: Inoculated** | **-7.11 (1.76)** | **-4.05** | **133.00** | **<0.001** |
|  | Sex: Male | -0.49 (1.00) | -0.49 | 133.00 | 0.63 |
|  | **Density: Tens x Inoculation: Inoculated** | **4.44 (2.13)** | **2.08** | **133.00** | **0.04** |

**Table S5.** Results of linear models investigating the effect of larval density on wing areas (forewing and hindwing), and melanin score in the food limitation experiment. Microcosm was included as a random effect in all linear models. Significant effect sizes (P-value ≤ 0.05) appear in bold.

| **Response Variable** | **Fixed Effect** | **Estimate (SE)** | **t value** | **d.f.** | **P-value** |
| --- | --- | --- | --- | --- | --- |
| Forewing area | **Density: Tens** | **-2.09 (0.23)** | **-8.95** | **83.17** | **<0.001** |
|  | Inoculation: Inoculated | -0.09 (0.27) | -0.34 | 131.72 | 0.73 |
|  | Sex: Male | 0.26 (0.15) | 1.73 | 131.44 | 0.08 |
|  | Density: Tens x Inoculation: Inoculated | 0.13 (0.33) | 0.41 | 82.56 | 0.68 |
| Hindwing area | **Density: Tens** | **-2.15 (0.24)** | **-9.07** | **80.15** | **<0.001** |
|  | Inoculation: Inoculated | -0.12 (0.28) | -0.47 | 132.65 | 0.64 |
|  | **Sex: Male** | **0.33 (0.16)** | **2.09** | **132.54** | **0.04** |
|  | Density: Tens x Inoculation: Inoculated | 0.09 (0.34) | 0.29 | 80.54 | 0.77 |
| Melanin score | **Density: Tens** | **6.00 (1.13)** | **5.30** | **64.20** | **<0.001** |
|  | **Inoculation: Inoculated** | **7.32 (1.26)** | **5.80** | **126.87** | **<0.001** |
|  | Sex: Male | -1.27 (0.72) | -1.80 | 129.00 | 0.07 |
|  | Density: Tens x Inoculation: Inoculated | 0.71 (1.63) | 0.43 | 65.12 | 0.67 |

**Table S6.** Results of linear models investigating the effect of larval density on (a) infection status, (b) spore load, size corrected spore load, and tolerance in the food limitation experiment. Microcosm was included as a random effect in all models. Significant effect sizes (P-value ≤ 0.05) appear in bold.

a)

| **Response Variable** | **Fixed Effect** | **Estimate (SE)** | **z value** | **P-value** |
| --- | --- | --- | --- | --- |
| Infection (0/1) | Density: Tens | 0.08 (0.66) | 0.12 | 0.91 |
|  | Sex: Male | -0.21 (0.60) | -0.36 | 0.72 |

b)

| **Response Variable** | **Fixed Effect** | **Estimate (SE)** | **t value** | **d.f.** | **P-value** |
| --- | --- | --- | --- | --- | --- |
| Spore load | **Density: Tens** | **-0.21 (0.10)** | **-2.10** | **16.83** | **0.05** |
|  | Sex: Male | -0.05 (0.09) | -0.59 | 45.83 | 0.56 |
| Size-corrected spore load | Density: Tens | -0.06 (0.13) | -0.53 | 17.05 | 0.60 |
|  | Sex: Male | -0.04 (0.04) | -1.02 | 188.54 | 0.31 |
| Tolerance  (adult lifespan) | Spore load | -5.20 (5.63) | -0.92 | 44.00 | 0.36 |
|  | Density: Tens | -19.41 (35.79) | -0.54 | 44.00 | 0.59 |
|  | Spore load x Density: Tens | 3.90 (6.60) | 0.58 | 44.00 | 0.56 |
|  | Sex: Male | 0.03 (1.85) | 0.02 | 44.00 | 0.99 |

**Table S7**. Post hoc pair-wise Tukey contrast results on probability of survival model (food unlimited experiment; Fig. 1A). Significant effect contrasts (P-value ≤ 0.05) appear in bold.

| **Contrast** | **Estimate (SE)** | **z value** | **P-value** |
| --- | --- | --- | --- |
| Doubles Control - Singles Control == 0 | -6.67e-02 (6.51e-02) | -1.02 | 0.91 |
| Tens Control - Singles Control == 0 | -1.02e-01 (5.83e-02) | -1.75 | 0.50 |
| Singles Inoculated - Singles Control == 0 | -1.20e-01 (6.78e-02) | -1.77 | 0.48 |
| Doubles Inoculated - Singles Control == 0 | -3.33e-02 (6.51e-02) | -0.51 | 1.00 |
| Tens Inoculated - Singles Control == 0 | -3.33e-02 (5.82e-02) | -0.57 | 0.99 |
| Tens Control - Doubles Control == 0 | -3.51e-02 (5.52e-02) | -0.64 | 0.99 |
| Singles Inoculated - Doubles Control == 0 | -5.33e-02 (6.51e-02) | -0.82 | 0.96 |
| Doubles Inoculated - Doubles Control == 0 | 3.33e-02 (6.23e-02) | 0.54 | 0.99 |
| Tens Inoculated - Doubles Control == 0 | 3.33e-02 (5.50e-02) | 0.61 | 0.99 |
| Singles Inoculated - Tens Control == 0 | -1.83e-02 (5.83e-02) | -0.31 | 1.00 |
| Doubles Inoculated - Tens Control == 0 | 6.84e-02 (5.52e-02) | 1.24 | 0.81 |
| Tens Inoculated - Tens Control == 0 | 6.84e-02 (4.67e-02) | 1.46 | 0.68 |
| Doubles Inoculated - Singles Inoculated == 0 | 8.67e-02 (6.51e-02) | 1.33 | 0.77 |
| Tens Inoculated - Singles Inoculated == 0 | 8.67e-02 (5.82e-02) | 1.49 | 0.67 |
| Tens Inoculated - Doubles Inoculated == 0 | 2.57e-16 (5.50e-02) | 0.00 | 1.00 |

**Table S8**. Post hoc pair-wise Tukey contrast results on total development model (food unlimited experiment; Fig. 1B). Significant effect contrasts (P-value ≤ 0.05) appear in bold.

| **Contrast** | **Estimate (SE)** | **z value** | **P-value** |
| --- | --- | --- | --- |
| Doubles Control - Singles Control == 0 | -0.10 (0.21) | -0.49 | 0.99 |
| Tens Control - Singles Control == 0 | -0.21 (0.18) | -1.14 | 0.86 |
| Singles Inoculated - Singles Control == 0 | 0.40 (0.22) | 1.83 | 0.44 |
| Doubles Inoculated - Singles Control == 0 | -0.05 (0.20) | -0.24 | 0.99 |
| Tens Inoculated - Singles Control == 0 | -0.29 (0.18) | -1.60 | 0.60 |
| Tens Control - Doubles Control == 0 | -0.11(0.18) | -0.62 | 0.99 |
| Singles Inoculated - Doubles Control == 0 | 0.50 (0.21) | 2.34 | 0.17 |
| Doubles Inoculated - Doubles Control == 0 | 0.05 (0.20) | 0.26 | 0.99 |
| Tens Inoculated - Doubles Control == 0 | -0.19 (0.18) | -1.08 | 0.89 |
| **Singles Inoculated - Tens Control == 0** | **0.61 (0.19)** | **3.18** | **0.02** |
| Doubles Inoculated - Tens Control == 0 | 0.16 (0.18) | 0.92 | 0.94 |
| Tens Inoculated - Tens Control == 0 | -0.08 (0.15) | -0.54 | 0.99 |
| Doubles Inoculated - Singles Inoculated == 0 | -0.45 (0.21) | -2.12 | 0.28 |
| **Tens Inoculated - Singles Inoculated == 0** | **-0.69 (0.19)** | **-3.63** | **<0.01** |
| Tens Inoculated - Doubles Inoculated == 0 | -0.24 (0.17) | -1.39 | 0.73 |

**Table S9.** Post hoc pair-wise Tukey contrast results on adult lifespan model (food unlimited experiment; Fig. 1C). Significant effect contrasts (P-value ≤ 0.05) appear in bold.

| **Contrast** | **Estimate (SE)** | **z value** | **P-value** |
| --- | --- | --- | --- |
| Doubles Control - Singles Control == 0 | -1.61 (1.13) | -1.42 | 0.71 |
| **Tens Control - Singles Control == 0** | **3.10 (1.10)** | **2.84** | **0.05** |
| **Singles Inoculated - Singles Control == 0** | **-9.03 (1.18)** | **-7.67** | **<0.001** |
| **Doubles Inoculated - Singles Control == 0** | **-11.14 (1.15)** | **-9.69** | **<0.001** |
| **Tens Inoculated - Singles Control == 0** | **-9.07 (1.09)** | **-8.34** | **<0.001** |
| **Tens Control - Doubles Control == 0** | **4.71 (1.08)** | **4.35** | **<0.001** |
| **Singles Inoculated - Doubles Control == 0** | **-7.42 (1.17)** | **-6.34** | **<0.001** |
| **Doubles Inoculated - Doubles Control == 0** | **-9.53 (1.15)** | **-8.34** | **<0.001** |
| **Tens Inoculated - Doubles Control == 0** | **-7.46 (1.08** | **-6.91** | **<0.001** |
| **Singles Inoculated - Tens Control == 0** | **-12.13 (1.13)** | **-10.73** | **<0.001** |
| **Doubles Inoculated - Tens Control == 0** | **-14.24 (1.10)** | **-12.91** | **<0.001** |
| **Tens Inoculated - Tens Control == 0** | **-12.17 (1.04)** | **-11.72** | **<0.001** |
| Doubles Inoculated - Singles Inoculated == 0 | -2.11 (1.19) | -1.78 | 0.48 |
| Tens Inoculated - Singles Inoculated == 0 | -0.04 (1.13) | -0.03 | 1.00 |
| Tens Inoculated - Doubles Inoculated == 0 | 2.07 (1.10) | 1.88 | 0.41 |

**Table S10.** Post hoc pair-wise Tukey contrast results on wing melanin score model (food unlimited experiment; Fig. 1D). Significant effect contrasts (P-value ≤ 0.05) appear in bold.

| **Contrast** | **Estimate (SE)** | **z value** | **P-value** |
| --- | --- | --- | --- |
| Doubles Control - Singles Control == 0 | -0.66 (0.73) | -0.90 | 0.95 |
| Tens Control - Singles Control == 0 | 0.66 (0.74) | 0.89 | 0.95 |
| Singles Inoculated - Singles Control == 0 | 1.38 (0.76) | 1.81 | 0.46 |
| **Doubles Inoculated - Singles Control == 0** | **3.00 (0.74)** | **4.04** | **<0.001** |
| **Tens Inoculated - Singles Control == 0** | **2.44 (0.74)** | **3.31** | **0.01** |
| Tens Control - Doubles Control == 0 | 1.32 (0.74) | 1.77 | 0.46 |
| Singles Inoculated - Doubles Control == 0 | 2.04 (0.77) | 2.66 | 0.08 |
| **Doubles Inoculated - Doubles Control == 0** | **3.66 (0.75)** | **4.91** | **<0.001** |
| **Tens Inoculated - Doubles Control == 0** | **3.10 (0.74)** | **4.19** | **<0.001** |
| Singles Inoculated - Tens Control == 0 | 0.72 (0.78) | 0.93 | 0.94 |
| **Doubles Inoculated - Tens Control == 0** | **2.34 (0.76)** | **3.10** | **0.02** |
| Tens Inoculated - Tens Control == 0 | 1.78 (0.75) | 2.38 | 0.16 |
| Doubles Inoculated - Singles Inoculated == 0 | 1.62 (0.78) | 2.09 | 0.29 |
| Tens Inoculated - Singles Inoculated == 0 | 1.06 (0.77) | 1.37 | 0.74 |
| Tens Inoculated - Doubles Inoculated == 0 | -0.56 (0.75) | -0.75 | 0.98 |

**Table S11.** Post hoc pair-wise Tukey contrast results on forewing area model (food unlimited experiment; Fig. 1E). Significant effect contrasts (P-value ≤ 0.05) appear in bold.

| **Contrast** | **Estimate (SE)** | **z value** | **P-value** |
| --- | --- | --- | --- |
| Doubles Control - Singles Control == 0 | -0.10 (0.17) | -0.60 | 0.99 |
| Tens Control - Singles Control == 0 | -0.18 (0.17) | -1.05 | 0.90 |
| Singles Inoculated - Singles Control == 0 | -0.16 (0.17) | -0.97 | 0.93 |
| Doubles Inoculated - Singles Control == 0 | -0.25 (0.17) | -1.48 | 0.68 |
| Tens Inoculated - Singles Control == 0 | -0.06 (0.17) | -0.38 | 1.00 |
| Tens Control - Doubles Control == 0 | -0.08 (0.17) | -0.46 | 1.00 |
| Singles Inoculated - Doubles Control == 0 | -0.06 (0.17) | -0.38 | 1.00 |
| Doubles Inoculated - Doubles Control == 0 | -0.15 (0.17) | -0.88 | 0.95 |
| Tens Inoculated - Doubles Control == 0 | 0.04 (0.17) | 0.21 | 1.00 |
| Singles Inoculated - Tens Control == 0 | 0.01 (0.18) | 0.08 | 1.00 |
| Doubles Inoculated - Tens Control == 0 | -0.07 (0.17) | -0.41 | 1.00 |
| Tens Inoculated - Tens Control == 0 | 0.11 (0.18) | 0.65 | 0.99 |
| Doubles Inoculated - Singles Inoculated == 0 | -0.08 (0.17) | -0.49 | 1.00 |
| Tens Inoculated - Singles Inoculated == 0 | 0.10 (0.17) | 0.58 | 0.99 |
| Tens Inoculated - Doubles Inoculated == 0 | 0.19 (0.17) | 1.07 | 0.89 |

**Table S12.** Post hoc pair-wise Tukey contrast results on hindwing area model (food unlimited experiment; Fig. 1F). Significant effect contrasts (P-value ≤ 0.05) appear in bold.

| **Contrast** | **Estimate (SE)** | **z value** | **P-value** |
| --- | --- | --- | --- |
| Doubles Control - Singles Control == 0 | -0.08 (0.18) | -0.43 | 1.00 |
| Tens Control - Singles Control == 0 | -0.13 (0.18) | -0.71 | 0.98 |
| Singles Inoculated - Singles Control == 0 | -0.19 (0.18) | -1.06 | 0.90 |
| Doubles Inoculated - Singles Control == 0 | -0.41 (0.18) | -2.30 | 0.19 |
| Tens Inoculated - Singles Control == 0 | -0.21 (0.18) | -1.16 | 0.86 |
| Tens Control - Doubles Control == 0 | -0.05 (0.18) | -0.29 | 1.00 |
| Singles Inoculated - Doubles Control == 0 | -0.12 (0.18) | -0.63 | 0.99 |
| Doubles Inoculated - Doubles Control == 0 | -0.34 (0.18) | -1.86 | 0.43 |
| Tens Inoculated - Doubles Control == 0 | -0.13 (0.18) | -0.73 | 0.98 |
| Singles Inoculated - Tens Control == 0 | -0.06 (0.19) | -0.34 | 1.00 |
| Doubles Inoculated - Tens Control == 0 | -0.28 (0.19) | -1.53 | 0.65 |
| Tens Inoculated - Tens Control == 0 | -0.08 (0.19) | -0.43 | 1.00 |
| Doubles Inoculated - Singles Inoculated == 0 | -0.22 (0.19) | -1.19 | 0.84 |
| Tens Inoculated - Singles Inoculated == 0 | -0.02 (0.19) | -0.09 | 1.00 |
| Tens Inoculated - Doubles Inoculated == 0 | 0.20 (0.19) | 1.11 | 0.88 |

**Table S13.** Post hoc pair-wise Tukey contrast results on proportion infected model (food unlimited experiment; Fig. 2A). Significant effect contrasts (P-value ≤ 0.05) appear in bold.

| **Contrast** | **Estimate (SE)** | **z value** | **P-value** |
| --- | --- | --- | --- |
| Doubles Inoculated - Singles Inoculated == 0 | 0.02 (0.07) | 0.22 | 0.97 |
| Tens Inoculated - Singles Inoculated == 0 | 0.07 (0.08) | 0.86 | 0.66 |
| Tens Inoculated - Doubles Inoculated == 0 | 0.05 (0.08) | 0.66 | 0.79 |

**Table S14.** Post hoc pair-wise Tukey contrast results on log_10_ parasite spore load model (food unlimited experiment; Fig. 2B). Significant effect contrasts (P-value ≤ 0.05) appear in bold.

| **Contrast** | **Estimate (SE)** | **z value** | **P-value** |
| --- | --- | --- | --- |
| Doubles Inoculated - Singles Inoculated == 0 | 0.19 (0.41) | 0.47 | 0.89 |
| Tens Inoculated - Singles Inoculated == 0 | 0.36 (0.43) | 0.84 | 0.68 |
| Tens Inoculated - Doubles Inoculated == 0 | 0.17 (0.43) | 0.39 | 0.92 |

**Table S15**. Post hoc pair-wise Tukey contrast results on survival model (food limited experiment Fig. 3A). Significant effect contrasts (P-value ≤ 0.05) appear in bold.

| **Contrast** | **Estimate (SE)** | **z value** | **P-value** |
| --- | --- | --- | --- |
| Tens Control - Singles Control == 0 | -3.53e-02 (9.85e-02) | -0.36 | 0.98 |
| Singles Inoculated - Singles Control == 0 | 1.49e-16 (1.08e-01) | 0.00 | 1.00 |
| Tens Inoculated - Singles Control == 0 | -1.17e-01 (9.83e-02) | -1.19 | 0.63 |
| Singles Inoculated - Tens Control == 0 | 3.53e-02 (9.85e-02) | 0.36 | 0.98 |
| Tens Inoculated – Tens Control == 0 | -8.19e-02 (8.81e-02) | -0.93 | 0.79 |
| Tens Inoculated - Singles Inoculated == 0 | -1.17e-01 (9.83e-02) | -1.19 | 0.63 |

**Table S16.** Post hoc pair-wise Tukey contrast results on total development model (food limited experiment Fig. 3B). Significant effect contrasts (P-value ≤ 0.05) appear in bold.

| **Contrast** | **Estimate (SE)** | **z value** | **P-value** |
| --- | --- | --- | --- |
| **Tens Control - Singles Control == 0** | **1.75 (0.65)** | **2.70** | **0.04** |
| Singles Inoculated - Singles Control == 0 | 0.36 (0.70) | 0.52 | 0.96 |
| Tens Inoculated - Singles Control == 0 | 0.83 (0.66) | 1.26 | 0.59 |
| Singles Inoculated - Tens Control == 0 | -1.39 (0.65) | -2.15 | 0.14 |
| Tens Inoculated - Tens Control == 0 | -0.92 (0.61) | -1.52 | 0.43 |
| Tens Inoculated - Singles Inoculated == 0 | 0.47 (0.66) | 0.71 | 0.89 |

**Table S17.** Post hoc pair-wise Tukey contrast results on adult lifespan model (food limited experiment, Fig. 3C). Significant effect contrasts (P-value ≤ 0.05) appear in bold.

| **Contrast** | **Estimate (SE)** | **z value** | **P-value** |
| --- | --- | --- | --- |
| **Tens Control - Singles Control == 0** | **-4.67 (1.50)** | **-3.13** | **0.01** |
| **Singles Inoculated - Singles Control == 0** | **-7.11 (1.76)** | **-4.05** | **< 0.001** |
| **Tens Inoculated - Singles Control == 0** | **-7.34 (1.51)** | **-4.86** | **< 0.001** |
| Singles Inoculated - Tens Control == 0 | -2.44 (1.49) | -1.64 | 0.35 |
| Tens Inoculated - Tens Control == 0 | -2.67 (1.20) | -2.22 | 0.12 |
| Tens Inoculated - Singles Inoculated == 0 | -0.23 (1.52) | -0.15 | 0.99 |

**Table S18**. Post hoc pair-wise Tukey contrast results on wing melanin score model (food limited experiment, Fig. 3D). Significant effect contrasts (P-value ≤ 0.05) appear in bold.

| **Contrast** | **Estimate (SE)** | **z value** | **P-value** |
| --- | --- | --- | --- |
| **Tens Control - Singles Control == 0** | **5.99 (1.13)** | **5.30** | **< 0.001** |
| **Singles Inoculated - Singles Control == 0** | **7.32 (1.26)** | **5.79** | **< 0.001** |
| **Tens Inoculated - Singles Control == 0** | **14.03 (1.16)** | **12.10** | **< 0.001** |
| Singles Inoculated - Tens Control == 0 | 1.32 (1.15) | 1.16 | 0.65 |
| **Tens Inoculated - Tens Control == 0** | **8.03 (1.04)** | **7.76** | **< 0.001** |
| **Tens Inoculated - Singles Inoculated == 0** | **6.71 (1.18)** | **5.69** | **< 0.001** |

**Table S19.** Post hoc pair-wise Tukey contrast results on forewing area model (food limited experiment, Fig. 3E). Significant effect contrasts (P-value ≤ 0.05) appear in bold.

| **Contrast** | **Estimate (SE)** | **z value** | **P-value** |
| --- | --- | --- | --- |
| **Tens Control – Singles Control == 0** | **-2.12 (0.24)** | **-9.01** | **< 0.001** |
| Singles Inoculated – Singles Control == 0 | -0.14 (0.27) | -0.50 | 0.96 |
| **Tens Inoculated – Singles Control == 0** | **-2.05 (0.24)** | **-8.59** | **< 0.001** |
| **Singles Inoculated – Tens Control == 0** | **1.99 (0.23)** | **8.57** | **< 0.001** |
| Tens Inoculated – Tens Control == 0 | 0.07 (0.19) | 0.38 | 0.98 |
| **Tens Inoculated – Singles Inoculated == 0** | **-1.92 (0.24)** | **-8.15** | **< 0.001** |

**Table S20.** Post hoc pair-wise Tukey contrast results on hindwing area model (food limited experiment, Fig. 3F). Significant effect contrasts (P-value ≤ 0.05) appear in bold.

| **Contrast** | **Estimate (SE)** | **z value** | **P-value** |
| --- | --- | --- | --- |
| **Tens Control – Singles Control == 0** | **-2.18 (0.24)** | **-9.09** | **< 0.001** |
| Singles Inoculated – Singles Control == 0 | -0.17 (0.28) | -0.62 | 0.93 |
| **Tens Inoculated – Singles Control == 0** | **-2.17 (0.24)** | **-8.94** | **< 0.001** |
| **Singles Inoculated – Tens Control == 0** | **2.01 (0.24)** | **8.37** | **< 0.001** |
| Tens Inoculated – Tens Control == 0 | 0.01 (0.20) | 0.03 | 1.00 |
| **Tens Inoculated – Singles Inoculated == 0** | **-2.00 (0.24)** | **-8.23** | **< 0.001** |

**Table S21.** Post hoc pair-wise Tukey contrast results on proportion infected model (food limited experiment, Fig. 4A). Significant effect contrasts (P-value ≤ 0.05) appear in bold.

| **Contrast** | **Estimate (SE)** | **z value** | **P-value** |
| --- | --- | --- | --- |
| Tens Inoculated - Singles Inoculated == 0 | 0.08 (0.66) | 0.12 | 0.91 |

**Table S22.** Post hoc pair-wise Tukey contrast results on log_10_ parasite spore load model (food limited experiment, Fig. 4B). Significant effect contrasts (P-value ≤ 0.05) appear in bold.

| **Contrast** | **Estimate (SE)** | **z value** | **P-value** |
| --- | --- | --- | --- |
| **Tens Inoculated - Singles Inoculated == 0** | **-0.21 (0.10)** | **-2.10** | **0.04** |
